# The Fight against Disease Mongering: Generating Knowledge for Action

**DOI:** 10.1371/journal.pmed.0030191

**Published:** 2006-04-11

**Authors:** Ray Moynihan, David Henry

## Abstract

The Guest Editors of the Disease Mongering theme issue argue that we need better research to identify, understand, and combat the threat to human health from the corporate-sponsored selling of sickness.


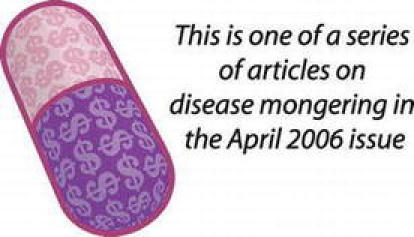


Disease mongering turns healthy people into patients, wastes precious resources, and causes iatrogenic harm. Like the marketing strategies that drive it, disease mongering poses a global challenge to those interested in public health, demanding in turn a global response. This theme issue of *PLoS Medicine* is explicitly designed to help provoke and inform that response.

## What Is Disease Mongering?

The problem of disease mongering is attracting increasing attention [ 1–3], though an adequate working definition remains elusive. In our view, disease mongering is the selling of sickness that widens the boundaries of illness and grows the markets for those who sell and deliver treatments. It is exemplified most explicitly by many pharmaceutical industry–funded disease-awareness campaigns—more often designed to sell drugs than to illuminate or to inform or educate about the prevention of illness or the maintenance of health. In this theme issue and elsewhere, observers have described different forms of disease mongering: aspects of ordinary life, such as menopause, being medicalised; mild problems portrayed as serious illnesses, as has occurred in the drug-company-sponsored promotion of irritable bowel syndrome (see pp. 156–174 in [ 2]; [ 4]) and risk factors, such as high cholesterol and osteoporosis, being framed as diseases.

Drug companies are by no means the only players in this drama. Through the work of investigative journalists, we have learned how informal alliances of pharmaceutical corporations, public relations companies, doctors' groups, and patient advocates promote these ideas to the public and policymakers—often using mass media to push a certain view of a particular health problem. While these different stakeholders may come to these alliances with different motives, there is often a confluence of interests—resulting in health problems routinely being framed as widespread, severe, and treatable with pills, as has happened recently with social anxiety disorder [ 5]. Currently, these alliances are working with the media to popularize little-known conditions, such as restless legs syndrome [ 6] and female sexual dysfunction [ 7], in each case lending credence to inflated prevalence estimates. In the case of female sexual dysfunction, there has been a serious, though heavily contested, attempt to convince the public in the United States that 43% of women live with this condition (see pp. 175–195 in [ 2]). This is happening at a time when pharmaceutical companies perceive a need to build and maintain markets for their big-selling products and when pipelines for new and genuinely innovative medicines are perceived as being weak.

“The coming years will bear greater witness to the corporate sponsored creation of disease.”

## A Context for Disease Mongering

Three decades ago, Ivan Illich argued polemically that the medical establishment was “medicalising” life itself [ 8], and in the 1990s Lynn Payer described widening the boundaries of illness as “disease mongering” [ 3], highlighting the role of pharmaceutical companies. Today's debate about this phenomenon, while still maturing, both acknowledges the axiomatic interest of corporations and professionals in maximizing turnover and appreciates that well-informed citizens may choose to embrace the medicalisation of health problems previously regarded as troublesome inconveniences.

It can also be argued that disease mongering is the opportunistic exploitation of both a widespread anxiety about frailty and a faith in scientific advance and “innovation”—a powerful economic, scientific, and social norm. In many nations, government policy priority is to secure market-based economic development, while more equitable social policies, such as public health strategies, can become subordinate or redundant. Disease mongering can thrive in such a normative environment. The practical consequences are that many of the so-called disease-awareness campaigns that inform our contemporary understanding of illness—whether as citizens, journalists, health professionals, industry leaders, academics, or policymakers—are now underwritten by the marketing departments of large drug companies rather than by organizations with a primary interest in public health. And it is no secret that those same marketing departments contract advertising agencies with expertise in “condition branding,” whose skills include “fostering the creation” of new medical disorders and dysfunctions [ 9]. As a recent *Reuters Business Insight* report on so-called lifestyle drugs— designed to be read by pharmaceutical industry leaders—pointed out, “The coming years will bear greater witness to the corporate sponsored creation of disease” [ 10]. We hope the coming years will also bear witness to a much more vigorous effort from within civil society to understand and to challenge that corporate process.

## Problems Defining Disease Mongering

While the term “disease mongering” is now commonly used as shorthand to describe campaigns that inappropriately widen the boundaries of treatable illness, there is uncertainty about how to operationally define the concept. With most disorders or conditions, there will be a number of individuals who suffer severe forms of the problem, who will benefit greatly from treatment and may be helped enormously by the publicity and marketing given to both the treatment and the disorder. For example, industry-funded awareness raising about the treatment and prevention of HIV/AIDS has surely been valuable. But in other cases, the same marketing/awareness-raising campaign will be viewed very differently depending on the perspective of the observer: what an industry-linked professional group may consider to be legitimate public education about an underdiagnosed disease, an activist group free from industry sponsorship may regard as a crude attempt to build markets for potentially dangerous drugs.

The Eli Lilly–sponsored promotion of premenstrual dysphoric disorder to help sell a re-branded version of fluoxetine (rebranded from Prozac to Sarafem) is a case in point (see pp. 99–118 in [ 2]). Considered by some as a serious psychiatric illness, premenstrual dysphoric disorder is regarded by others as a condition that does not exist.

These discordant views of the same activity reinforce the fact that there are often different motives for the different individuals who get caught up in disease-mongering campaigns. In the pharmaceutical industry and in the public relations companies that serve them, the marketers often now dominate. But these corporations are not heterogeneous, and staff working in research or medical departments may express the same doubts as many working outside industry. For their part, the motives of health professionals and health advocacy groups may well be the welfare of patients, rather than any direct self-interested financial benefit, but we believe that too often marketers are able to crudely manipulate those motivations. Disentangling the different motivations of the different actors in disease mongering will be a key step towards a better understanding of this phenomenon.

## Generating Better Knowledge

The views in this article are based on observations and interpretation informed by interviews with stakeholders and other more journalistic research methods, rather than a deeper academic investigation that employs qualitative and quantitative research techniques. Before embarking on research agendas to investigate disease mongering and its impacts, a broader conception of the phenomenon is warranted—requiring researchers to explore the uncertainty surrounding the definition of the problem, how and why different stakeholders understand it differently, and the deeper social and economic contexts. For example, the broad shift away from government-run programs and towards the marketplace within social democracies worldwide, and the consequent commercialisation and commodification of health services, may be a useful framework for a more profound explanation of this problem. In a climate where governments are encouraging corporations to vigorously pursue for-profit activities within the health-care sector, it is hardly surprising that pharmaceutical companies will use a range of promotional activities to widen the definitions of disease in order to grow the potential markets for their products. [Fig pmed-0030191-g001]

**Figure pmed-0030191-g001:**
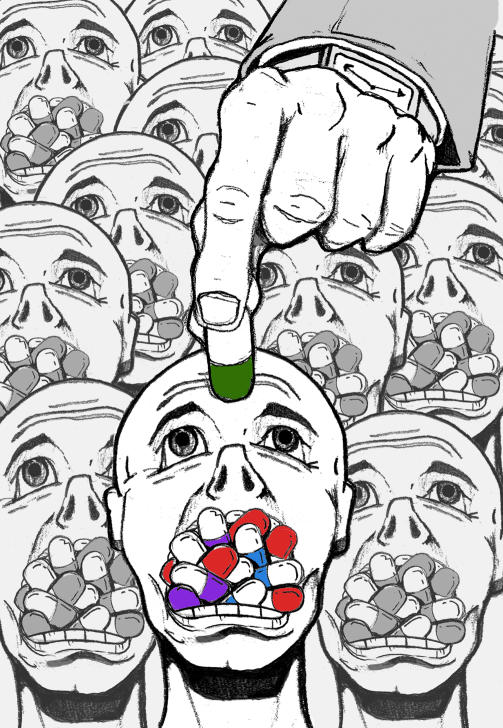
Most people in Western countries take medication to treat or prevent illness or enhance well-being (Illustration: Anthony Flores)

Along with deeper reflection, we suggest researchers start to develop strategies for generating data on the impact of disease mongering. More conventional health-science methodologies may prove to be valuable ways of investigating the potential influences of a disease-marketing campaign on outcomes such as public perceptions of a particular disease, prevalence/incidence rates for that disease, prescription patterns for the drugs linked to that disease, and even health status of those diagnosed with and/or treated for that disease. Multisite controlled studies of drug company–funded disease-awareness campaigns would be the ideal. However, defining appropriate control groups and devising indices to measure outcomes such as inappropriate medicalisation will prove extremely challenging since almost everyone is exposed to disease mongering in some form. Similarly, rigorous studies of publicly funded “counter-detailing”—where noncommercially oriented information about disease is promoted to physicians and citizens—may be warranted, though, again, it is very difficult methodologically.

A challenge to the excesses of disease mongering may come from within the industry.

Apart from these more challenging approaches, we believe there is a range of research projects that are both achievable and urgently needed. First, academic investigation of the prevalence of this problem would be highly desirable. Researchers could, for example, take a group of the most common (high-burden) diseases/conditions, and investigate how and why the definitions of those diseases/conditions have changed over time in different nations. Such retrospective investigations could include analysis of the decisions and recommendations of the panels that define and redefine illness, the evidence informing those decisions, the conflicts of interest of panel members and their respective professional bodies, and the sponsorship of these processes. Early versions of this investigation are happening in a random, ad hoc way [ 11], but a coordinated systematic effort by a multinational group of respected researchers or research institutes is obviously preferable. As part of such an examination, a series of case studies would inevitably emerge, warranting deeper study and research and serving as a way to popularize awareness of the process of disease mongering.

Another potentially rich research method might involve a prospective study of the launch of a new or recently expanded disease or condition. A global collaboration could, for example, study the way female sexual dysfunction is being constructed and then promoted. “Creating the need” is now an established and integral part of the promotion of any new blockbuster drug, and sometimes that involves introducing a whole new condition to the wider public [ 12]. The success of sildenafil depended on corporate-funded disease-awareness campaigns promoting erectile dysfunction [ 13], and similarly the commercial success of any pharmaceutical treatments for female sexual dysfunction will hang in part on similar campaigns. While activists and scholars have begun the process of observing these activities, it is our view that the magnitude of public and private resources spent on these products, the potential harm that can flow from inappropriate medicalisation, and the opportunity cost in terms of treating and preventing genuine pathology demands more rigorous scientific investigation.

## Time for Action?

Around the world, there are tentative steps to identify, understand, and combat the threat to human health from the corporate-sponsored selling of sickness. These small steps are being taken by several players within the health field, and we trust this theme issue may support and augment these developments.

At a consumer level, Health Action International ( http://www.haiweb.org)—the activist group working for a more rational use of medicines globally—has for a long time been concerned about what it has described as the blurring of boundaries between ordinary life and medical illness in order to expand markets for drugs and other technologies [ 14]. Unlike many patient advocacy groups, Health Action International does not accept pharmaceutical company sponsorship, and actively warns others about the threats to independence from doing so [ 15]. By way of contrast, many consumer/advocacy groups around the world now rely on such funding [ 16], raising questions about their credibility, particularly as they are often used as the human face of disease-awareness campaigns sponsored by their funders. An open debate within the health consumer movement about its close engagement with industry, and its involvement in disease mongering, would be welcome.

Likewise, amongst journalist circles, there are nascent debates about the media's propensity to exaggerate disease prevalence and severity, and how to deal with this problem. In this issue of *PLoS Medicine*, two high-profile scholars with an interest in the area of medicine and the media, Lisa Schwartz and Steven Woloshin, present a timely and relevant case study on the “selling” of restless legs syndrome [ 6]. In Australia and Canada, a new media watch group called Media Doctor is also investigating the extent to which media stories on medicine either report appropriately on the nature and extent of illness or tend to simply regurgitate the promotional messages of disease-mongering campaigns ( http://www.mediadoctor.org.au).

While many professional organizations remain reliant on industry support, some are actively debating the problem of disease mongering. In a submission to the recent House of Commons inquiry into the influence of the pharmaceutical industry in Britain, the Royal College of General Practitioners outlined serious concerns about the process [ 17]. The subsequent report recommended that industry-funded disease-awareness campaigns should no longer be “veiled advertising” of branded drugs [ 18].

Shareholders in the world's large pharmaceutical companies have the strongest financial interest in widening the boundaries of treatable illness in order to widen markets for their products. Yet in the debate about research and development for treatments for neglected diseases in the developing world, there are strong signs that shareholders can support policies driven by motivations other than profit [ 19]. It may be that as key shareholders and company executives alike understand more of the implications of what their marketing departments do, a challenge to the excesses of disease mongering may come from within industry, just as other parts of the health sector challenge excesses of disease mongering from within.

## Conclusion

Genuine sustainable change, however, will not come until policymakers better understand the phenomenon of disease mongering and the potential benefits of responding against it. In Australia, for example, it has been estimated that winding back the public subsidy for inappropriate prescriptions of several high-profile drugs to people with milder health problems could save hundreds of millions of dollars per year [ 20]. Those responsible for managing Australia's publicly funded national formulary, the Pharmaceutical Benefits Scheme, have become increasingly concerned about what is described as “leakage”—the process where subsidised drugs are prescribed by physicians to people for whom use of the drug has been deemed not cost-effective because of a poor cost–benefit ratio. We suspect that the estimated hundreds of millions of dollars of public money wasted on leakage in Australia annually is in part a result of drug companies promoting their products, through physicians, to people with mild problems for whom a powerful prescription may be unnecessary or even do more harm than good. In summary, combating disease mongering may improve the personal health of individuals, as well as the financial health of public (and private) insurers.

As an initial step toward combating disease mongering at a health policy level, we would urge decision makers to promote a renovation in the way diseases are defined. Continuing to leave these definitions to panels of self-interested specialists riddled with professional and commercial conflicts of interest is no longer viable. As a priority, new panels should be assembled, free of commercial conflicts of interest, involving a much wider, and less self-interested, group of players, who would ultimately generate more credible information.

Until a rigorous research agenda is initiated, and the social renovations and policy reforms that research might inform are enacted and evaluated, our beliefs, like those who argue for the benefits of corporate-sponsored disease-awareness campaigns, will remain based more on opinion than evidence. We hope this theme issue can start to change that.
